# Supplementary figures and images for: A pilot study: Auditory steady-state responses (ASSR) can be measured in human fetuses using fetal magnetoencephalography (fMEG)
Source: PLoS One. 2020 Jul 22;15(7):e0235310. doi: 10.1371/journal.pone.0235310 (PMC7375519; doi:10.1371/journal.pone.0235310)

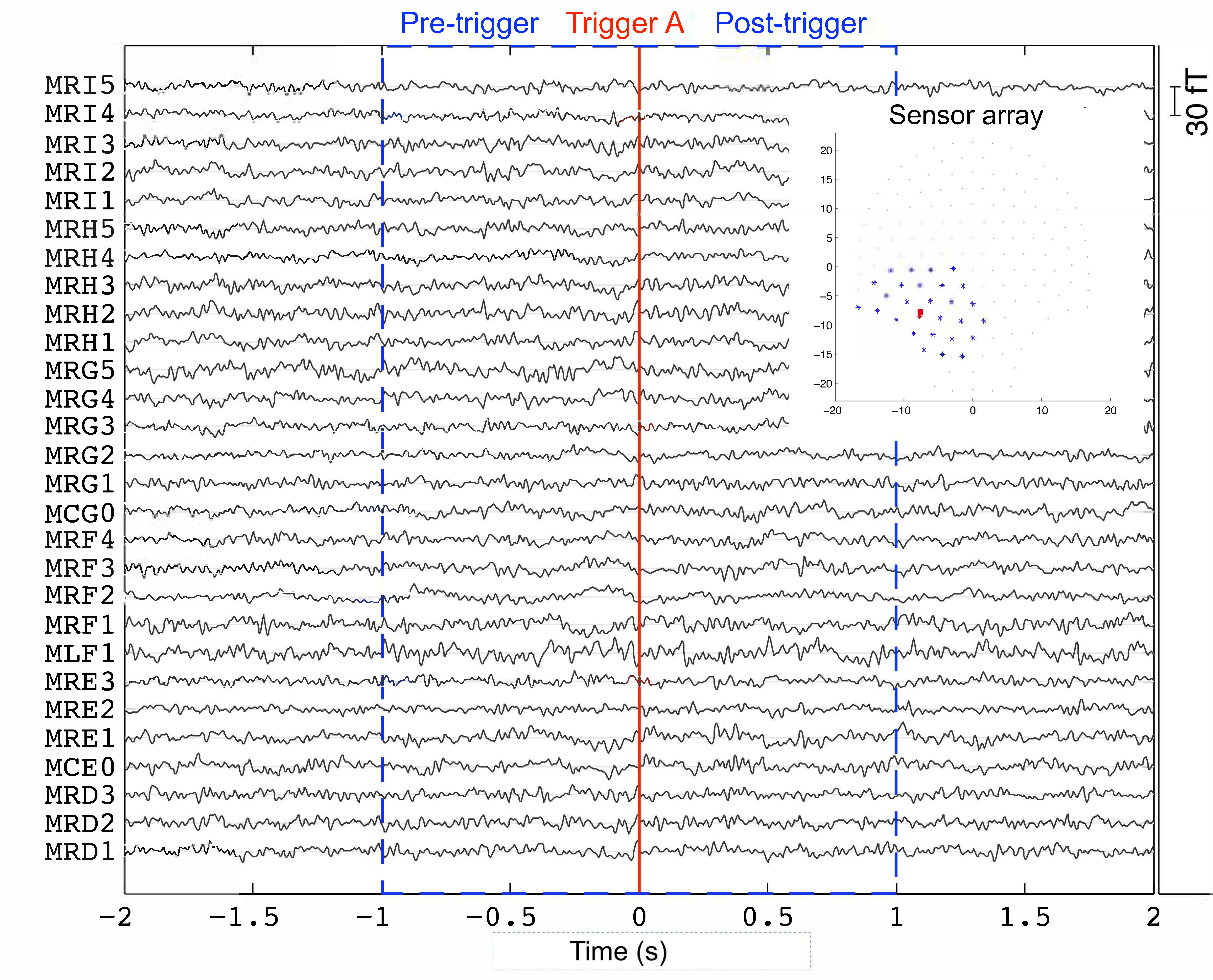

Supplement: S1 Fig — This figure shows the fMEG data of an exemplary recording called ‘SS27_35w0d’. A bandpass filter was applied at 1–50 Hz, maternal and fetal cardiograms were removed, and the data was averaged over all trails. The names on the y-axis represent individual sensors, the x-axis shows time in relation to the ‘Trigger A’ marker. Trigger A (red line) and pre-trigger or post-trigger data (blue, dashed line) are indicated. The subfigure in the right upper corner shows all selected sensors (blue stars) within a 10 cm radius from the head coil (red square) on the SARA sensor array. Sensors that were not selected for analysis are represented by gray dots. (TIFF) [file pone.0235310.s001.tiff]
