# Supplementary material for: A pilot study: Auditory steady-state responses (ASSR) can be measured in human fetuses using fetal magnetoencephalography (fMEG)
Source: PLoS One. 2020 Jul 22;15(7):e0235310. doi: 10.1371/journal.pone.0235310 (PMC7375519; doi:10.1371/journal.pone.0235310)
Supplement: S1 Table — This table summarizes the criteria used in this study to define fetal behavioral states based on a visual analysis of the fetal actocardiogram. (DOCX) [file pone.0235310.s002.docx]

### S1 Table: Criteria for the definition of fetal behavioral states. This table summarizes the criteria used in this study to define fetal behavioral states based on a visual analysis of the fetal actocardiogram.


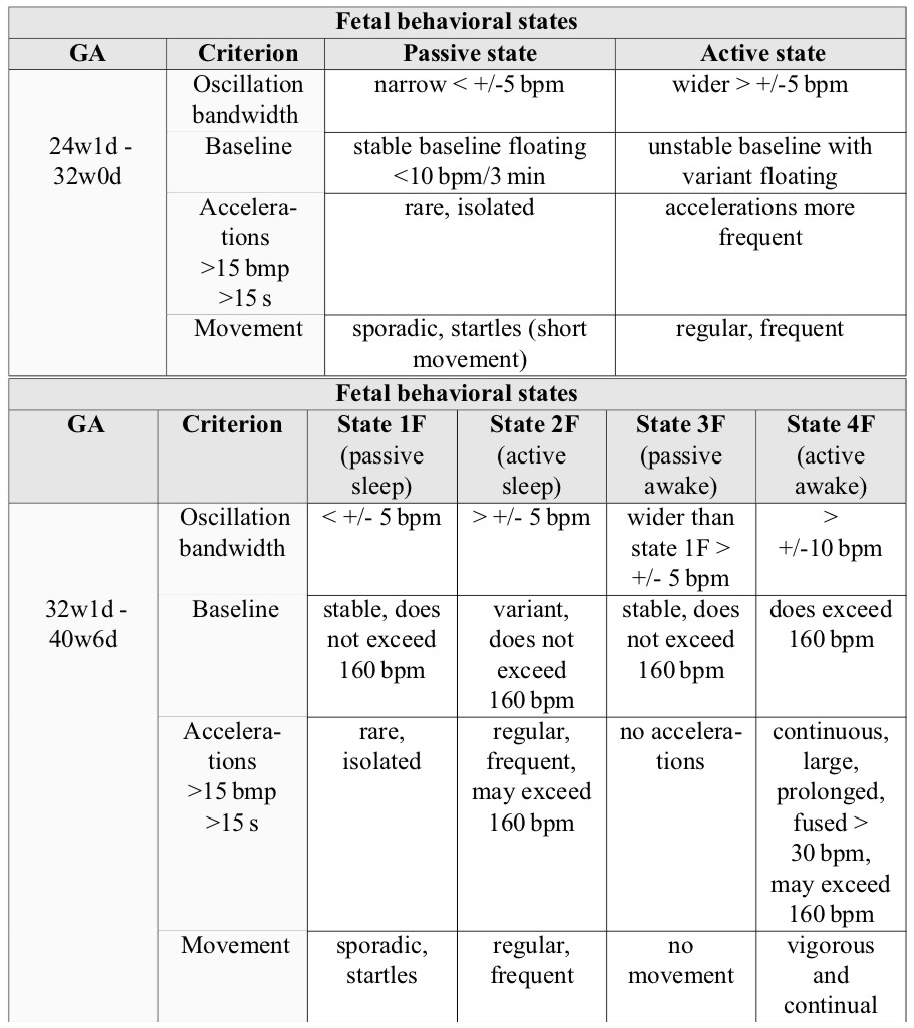


**Table S1.** Criteria used for the definition of fetal behavioral states based on the visual evaluation of the fetal actocardiogram. A state was only defined it a pattern lasted for at least three minutes. bpm stands for beats per minute. Source: MEG Center Tübingen
