# Supplementary material for: A pilot study: Auditory steady-state responses (ASSR) can be measured in human fetuses using fetal magnetoencephalography (fMEG)
Source: PLoS One. 2020 Jul 22;15(7):e0235310. doi: 10.1371/journal.pone.0235310 (PMC7375519; doi:10.1371/journal.pone.0235310)
Supplement: S2 Table — This table provides the exact values of the statistical analysis including the calculated standard error and p-values. (DOCX) [file pone.0235310.s003.docx]

**S2 Table: Overall group analysis for the MF of 27 Hz.** This table provides the exact values of the statistical analysis including the calculated standard error and p-values.

| **Least Squares Means** | | | | | | | | | |
| --- | --- | --- | --- | --- | --- | --- | --- | --- | --- |
| **Effect** | **reclabel** | **Estimate** | **Standard Error** | **DF** | **t Value** | **Pr > t** | **Alpha** | **Lower** | **Upper** |
| **reclabel** | NegControl:  measured value  (standardized)^†^ | 0.000601  (0.028 SDs) | 0.003325  (0.155 SDs) | 52 | 0.18 | 0.4286 | 0.05 | -0.004970  (-0.231 SDs) | 0.006170  (0.287 SDs) |
| **reclabel** | Stimulus:  measured value  (standardized)^†^ | 0.006227  (0.290 SDs) | 0.003325  (0.155 SDs) | 52 | 1.87 | 0.0335 | 0.05 | 0.000659  (0.031 SDs) | 0.01180  (0.549 SDs) |

†Standardized values were computed from measured values using an estimated SD of 0.021494

**Table S2.** Results of the statistical group analysis for all recordings using the MF of 27 Hz. The estimated post-trigger minus pre-trigger value (‘Estimate’), the corresponding standard error, the degree of freedom (‘DF’), the t-value (‘t Value’), the one-sided p-value in the positive direction (‘Pr *>* t’), the significance level (‘Alpha’) and the upper and lower limit of the 90% intervals (‘Upper’ and ‘Lower’) are displayed for stimulation recordings and negative controls.
