# Supplementary material for: A pilot study: Auditory steady-state responses (ASSR) can be measured in human fetuses using fetal magnetoencephalography (fMEG)
Source: PLoS One. 2020 Jul 22;15(7):e0235310. doi: 10.1371/journal.pone.0235310 (PMC7375519; doi:10.1371/journal.pone.0235310)
Supplement: S3 Table — This table provides the exact values of the statistical analysis including the calculated standard error and p-values. (DOCX) [file pone.0235310.s004.docx]

**S3 Table: Overall group analysis for the MF of 42 Hz.** This table provides the exact values of the statistical analysis including the calculated standard error and p-values.

| **Least Squares Means** | | | | | | | | | |
| --- | --- | --- | --- | --- | --- | --- | --- | --- | --- |
| **Effect** | **reclabel** | **Estimate** | **Standard Error** | **DF** | **t Value** | **Pr > t** | **Alpha** | **Lower** | **Upper** |
| **reclabel** | NegControl:  measured value  (standardized)^†^ | -0.00139  (-0.077 SDs) | 0.003152  (0.175 SDs) | 52 | -0.44 | 0.6699 | 0.05 | -0.00667  (-0.369 SDs) | 0.003884  (0.215 SDs) |
| **reclabel** | Stimulus:  measured value  (standardized)^†^ | -0.00106  (-0.059 SDs) | 0.003152  (0.175 SDs) | 52 | -0.34 | 0.6312 | 0.05 | -0.00634  (-0.351 SDs) | 0.004217  (0.234 SDs) |

†Standardized values were computed from measured values using an estimated SD of 0.018055

**Table S3.** Results of the statistical group analysis for all recordings using the MF of 42 Hz. The estimated post-trigger minus pre-trigger value (‘Estimate’), the corresponding standard error, the degree of freedom (‘DF’), the t-value (‘t Value’), the one-sided p-value in the positive direction (‘Pr *>* t’), the significance level (‘Alpha’) and the upper and lower limit of the 90% intervals (‘Upper’ and ‘Lower’) are displayed for stimulation recordings and negative controls.

### 
