# Supplementary material for: A pilot study: Auditory steady-state responses (ASSR) can be measured in human fetuses using fetal magnetoencephalography (fMEG)
Source: PLoS One. 2020 Jul 22;15(7):e0235310. doi: 10.1371/journal.pone.0235310 (PMC7375519; doi:10.1371/journal.pone.0235310)
Supplement: S4 Table — This table provides the exact values of the statistical analysis including the calculated standard error and p-values. (DOCX) [file pone.0235310.s005.docx]

**S4 Table:** **Subgroup analysis according to gestational age group for the MF of 27 Hz.** This table provides the exact values of the statistical analysis including the calculated standard error and p-values.

| **Estimates** | | | | | | | | |
| --- | --- | --- | --- | --- | --- | --- | --- | --- |
| **Label** | **Estimate** | **Standard Error** | **DF** | **t Value** | **Pr > t** | **Alpha** | **Lower** | **Upper** |
| **NegControl, Early** | 0.005417 | 0.006524 | 48 | 0.83 | 0.2053 | 0.05 | -0.00553 | 0.01636 |
| **NegControl, Late** | -0.00534 | 0.006252 | 48 | -0.85 | 0.8012 | 0.05 | -0.01582 | 0.005150 |
| **NegControl, Middle** | 0.001974 | 0.005798 | 48 | 0.34 | 0.3675 | 0.05 | -0.00775 | 0.01170 |
| **NegControl, Avg.** | 0.000685 | 0.003382 | 48 | 0.20 | 0.4202 | 0.05 | -0.00499 | 0.006357 |
| **Stimulus, Early** | 0.004940 | 0.006524 | 48 | 0.76 | 0.2263 | 0.05 | -0.00600 | 0.01588 |
| **Stimulus, Late** | 0.009696 | 0.006252 | 48 | 1.55 | 0.0638 | 0.05 | -0.00079 | 0.02018 |
| **Stimulus, Middle** | 0.004332 | 0.005798 | 48 | 0.75 | 0.2293 | 0.05 | -0.00539 | 0.01406 |
| **Stimulus, Avg.** | 0.006323 | 0.003382 | 48 | 1.87 | 0.0339 | 0.05 | 0.000650 | 0.01200 |

**Table S4.** Results of the subgroup analysis according to gestational age groups using the MF of 27 Hz. The estimated post-trigger minus pre-trigger value (‘Estimate’), the corresponding standard error, the degree of freedom (‘DF’), the t-value (‘t Value’), the one-sided p-value in the positive direction (‘Pr *>* t’), the significance level (‘Alpha’) and the upper and lower limit of the 90% intervals (‘Upper’ and ‘Lower’) are displayed for stimulation recordings and negative controls.
